# Genetic lineage tracing defines distinct neurogenic and gliogenic stages of ventral telencephalic radial glial development

**DOI:** 10.1186/1749-8104-3-30

**Published:** 2008-11-05

**Authors:** Todd E Anthony, Nathaniel Heintz

**Affiliations:** 1 Laboratory of Molecular Biology, the Rockefeller University, 1230 York Avenue, New York, New York 10021, USA; 2 Howard Hughes Medical Institute, the Rockefeller University, 1230 York Avenue, New York, New York 10021, USA

## Abstract

**Background:**

Radial glia comprise a molecularly defined neural progenitor population but their role in neurogenesis has remained contested due to the lack of a single universally accepted genetic tool for tracing their progeny and the inability to distinguish functionally distinct developmental stages.

**Results:**

By direct comparisons of Cre/*loxP *lineage tracing results obtained using three different radial glial promoters (*Blbp*, *Glast*, and *hGFAP*), we show that most neurons in the brain are derived from radial glia. Further, we show that *hGFAP *promoter induction occurs in ventral telencephalic radial glia only after they have largely completed neurogenesis.

**Conclusion:**

These data establish the major neurogenic role of radial glia in the developing central nervous system and genetically distinguish an early neurogenic *Blbp*^+^*Glast*^+^*hGFAP*^- ^stage from a later gliogenic *Blbp*^+^*Glast*^+^*hGFAP*^+ ^stage in the ventral telencephalon.

## Background

Radial glia (RG) comprise a molecularly defined cellular population, playing critical roles during central nervous system (CNS) development as both neural progenitors and as a scaffolding for migrating neurons [1,2]. Despite their established role as the major neuronal precursors in the cerebral cortex [3-8], two fundamental aspects of RG development and function remain contested. The first regards the question of whether, similar to the situation in cortex, RG function as the principal neuronal progenitors in all CNS regions. This question has been addressed with Cre/*loxP *lineage tracing using two different RG-specific promoters, the mouse brain lipid binding protein (*Blbp*) promoter and the human glial fibrillary acidic protein (*hGFAP*) promoter. However, sharply different patterns of recombination were driven by each promoter, prompting two distinct models for the RG role in neurogenesis. As significant neuronal recombination was detected in only a subset of brain regions when the *hGFAP *promoter was used to drive Cre [5,9], it was proposed that RG differ regionally in terms of their neurogenic potential and generate neurons only in certain brain regions [5,10,11]. In contrast, the extensive recombination driven in all brain regions by the *Blbp *promoter supported an alternative model in which essentially all RG populations are neurogenic and generate most neurons in the CNS [8]. Each model has different implications not only for RG function but also the larger issue of the mechanisms used to generate neurons during development. An accurate picture of lineal relationships within the developing brain is, therefore, essential but the controversy surrounding the RG contribution to neurogenesis has remained unresolved.

A second related issue of contention concerns the question of when cells with the molecular properties of RG first appear in the CNS. Resolution of this question is necessary as elucidation of the mechanisms responsible for driving RG differentiation requires analysis of the relevant pathways when they first become active, not at later stages when the end products of these programs become morphologically evident. Two different estimates for the time point when induction of RG differentiation occurs in the murine forebrain have been proposed based on Cre/*loxP *lineage tracing results (used to indicate onset of transcription). Whereas the mouse *Blbp *promoter drove recombination from embryonic day (E)10 [8], the *hGFAP *promoter is not active until after E12 [10]. These contrasting results have been alternatively proposed to reflect either precocious or delayed activity of the *Blbp *or *hGFAP *promoters, respectively [8,10,11], but neither direct evidence supporting either model nor data obtained using an independent RG promoter has yet been reported.

A third deficiency in the current understanding of RG development is the inability to distinguish RG at distinct stages of maturation. Multiple lines of evidence indicate that RG populations progress stepwise through distinct developmental stages during which particular classes of neurons or glia are generated, and that progression to successively later stages is accompanied by restricted potential to produce earlier cell types. For example, cortical RG stop generating neurons at late stages of embryogenesis and transform into astrocytes postnatally [1]. Determination of the precise time points when such transitions occur using *in vivo *genetic lineage tracing is a prerequisite to the identification of mechanisms regulating shifts in neurogenic potential; to date, few such studies have been conducted focusing on RG.

We report here that: similar to what was observed using the *Blbp *promoter for lineage tracing, RG expressing the glial high affinity glutamate transporter (*Glast, a universally accepted RG marker *[10-12]) are molecularly detectable in the forebrain by E10 and generate most neurons in the brain; *hGFAP *promoter activity is poorly correlated with RG differentiation in multiple brain regions, failing to drive recombination until days after RG first appear; and in ventral telencephalon, *hGFAP *activity commences after RG are predominantly gliogenic, genetically distinguishing an early neurogenic *Blbp*^+^*Glast*^+^*hGFAP*^- ^stage from a later gliogenic *Blbp*^+^*Glast*^+^*hGFAP*^+ ^stage in this region.

## Results

### Endogenous GLAST expression and recombination in *Glast*::Cre;R26R mice are detected in the forebrain from E10.5

Due to the discrepant results previously obtained using the *hGFAP *and *Blbp *promoters, we performed Cre/*loxP *lineage tracing using the *Glast *promoter to drive Cre expression. Like *Blbp*, *Glast *expression is restricted to RG and astrocytes and molecularly distinguishes them from developmentally earlier neuroepithelial stem cells [5,12,13]. To generate mice expressing Cre under the regulation of the *Glast *promoter, we inserted a Cre-polyA cassette into the second exon of the *Glast *gene in a bacterial artificial chromosome (BAC) and generated BAC transgenic mice. Five separate *Glast*::Cre founder lines were obtained, all of which yielded similar results when used for lineage tracing; representative results from one line are presented.

**Figure 1:**
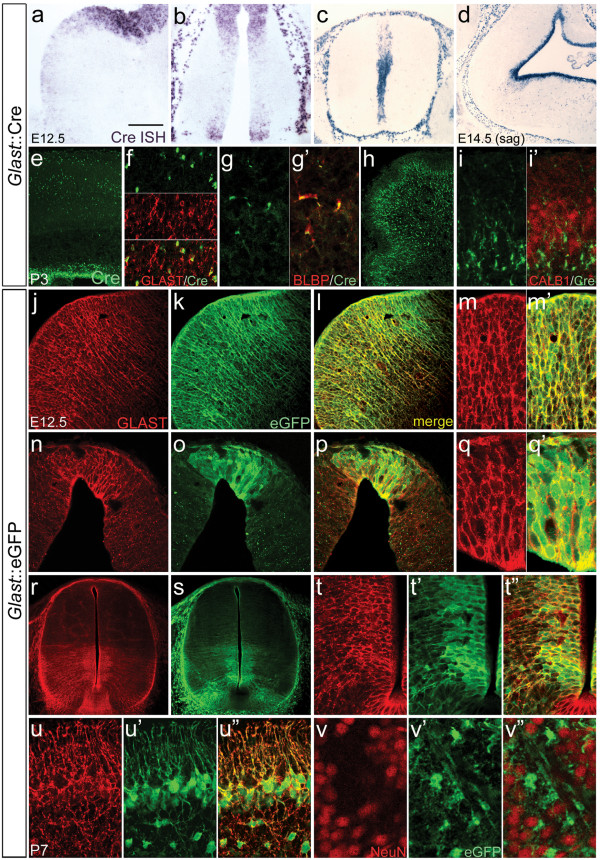
***Glast *bacterial artificial chromosome (BAC) transgenic mice recapitulate the spatiotemporal pattern of endogenous GLAST (glial high affinity glutamate transporter) expression.** (e-v") *In situ *hybridization (ISH) (a-d) and immunofluorescent staining (e-v) of *Glast*::Cre (a-i) and *Glast*::eGFP (j-v) BAC transgenic mice. Cre mRNA in the ganglionic eminences (a), thalamus (b), spinal cord (c), and cerebellum/hindbrain (d) was completely restricted to the ventricular zone, with no neuronal expression detected at any age examined. The *Glast*::Cre transgene also recapitulated endogenous GLAST expression gradients (compare (c) and (r)). Postnatally, Cre immunoreactivity (green) (e-i) was restricted to astroglia. Shown are cortical GLAST^+ ^astrocytes (f), striatal BLBP^+ ^astrocytes (g), and cerebellar Bergmann and astroglia (h,i); no colocalization with CALB1^+ ^Purkinje cells was observed (i). Double immunofluorescent staining of *Glast*::eGFP embryos for GLAST (red) and enhanced green fluorescent protein (eGFP; green) showed that the BAC transgene drives expression in essentially all GLAST^+ ^radial glia (j-q); the notable exception to this was the spinal cord floorplate where no transgene expression was detectable (r-t). Postnatal expression was similarly restricted to astroglia (u.v). Scale bars: 200 μm (c,d,e,h,r,s); 100 μm (b); 75 μm (a,j-l); 50 μm (f,n-p,t); 40 μm (m); 35 μm (u); 25 μm (g,i,q,v). E, embryonic day.

Specificity of the *Glast *BAC was confirmed using *in situ *hybridization (ISH) and immunofluorescent staining. Cre mRNA was detected in ventricular zone (VZ) cells throughout the embryonic CNS and closely paralleled endogenous *Glast *expression (Figure 1a–d). Importantly, all *Glast*-expressing regions contained Cre mRNA and regions known to differentially express GLAST showed similar Cre mRNA gradients (Figure 1c,r). Postnatally, Cre protein was absolutely restricted to astroglial cells in all brain regions examined; no colocalization with neuronal markers was observed (Figure 1e–i). To confirm that the *Glast *BAC drives specific expression in all GLAST^+ ^RG, *Glast*::eGFP transgenics using the same BAC vector and marker insertion locus were analyzed. Immunostaining demonstrated specific enhanced green fluorescent protein (eGFP) expression in the majority of GLAST^+ ^RG and astrocytes, endogenous GLAST gradients were faithfully recapitulated, and no neuronal expression was detectable (Figure 1j–v). Taken together, these data establish that the *Glast*::Cre transgene drives RG- and astroglial-specific expression and can be used to trace the neuronal progeny of all *Glast*-expressing progenitors in the brain. It should be noted however that BAC-driven expression was absent from the spinal cord floorplate (Figure 1r–t); as such, progeny derived from floorplate *Glast*^+ ^progenitors can not be traced in these mice.

**Figure 2:**
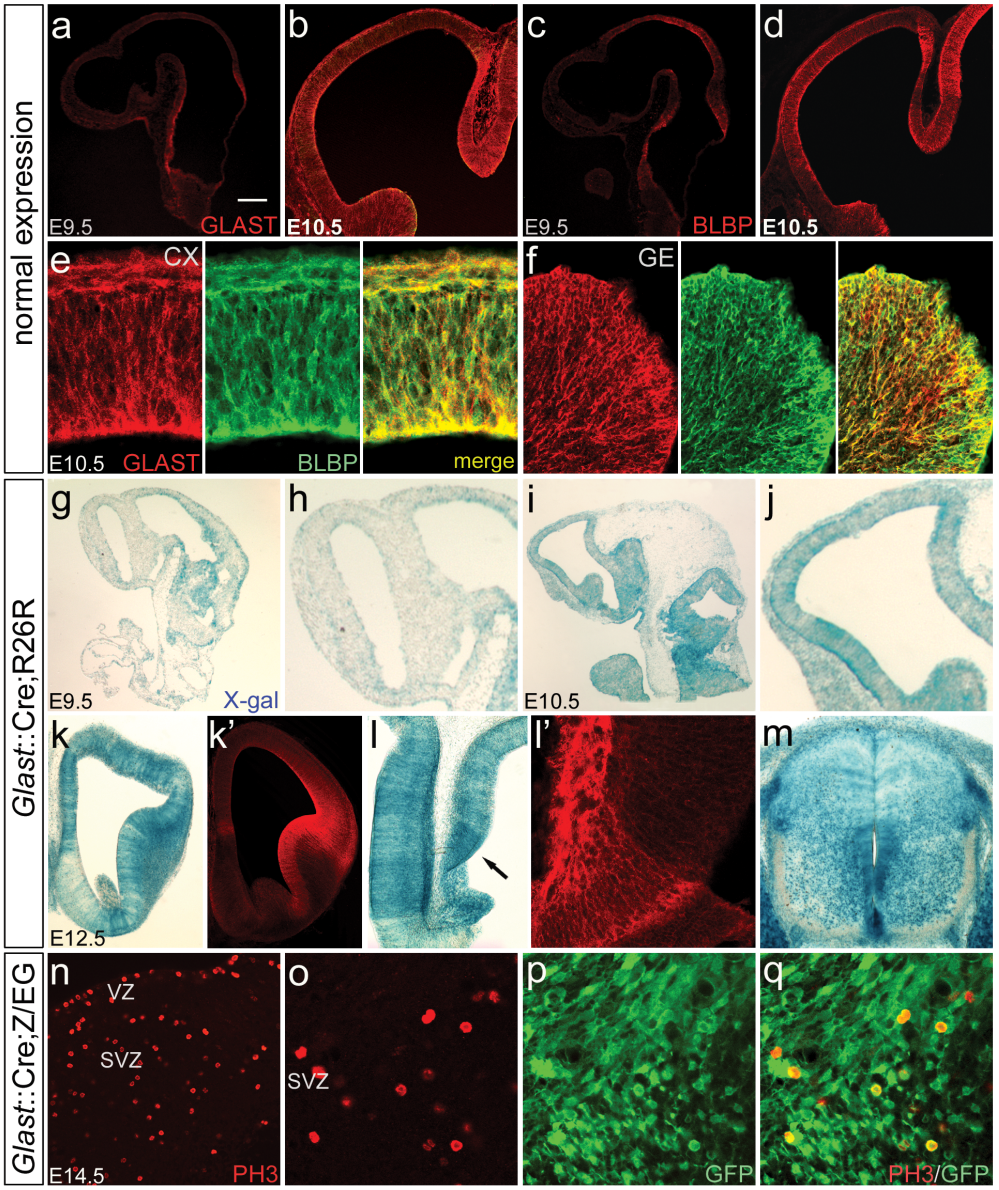
**Recombination in *Glast*::Cre;R26R embryos parallels endogenous GLAST (glial high affinity glutamate transporter) expression and the pattern observed in *Blbp*::Cre;R26R embryos.** (a-q) Wild-type (a-f), *Glast*::Cre;R26R^lacZ ^(g-m) and *Glast*::Cre;Z/EG (n-q) embryos immunostained for GLAST (a,b,e,f,k,l), BLBP (c-f), phosphorylated histone H3 (PH3) (n,o,q) or GFP (p,q) and X-gal staining (g-j,k',l',m'). Sections in (a-j) are sagittal; (k-q) are coronal. At E9.5, endogenous GLAST (a) and BLBP (c) expression was detected in hindbrain and spinal cord, but not in telencephalon until E10.5 (b,d). Double labeling for GLAST (red) and BLBP (green) in E10.5 CX and GE confirmed that both are upregulated in the same cells (e,f). The onset of Cre recombination matched the spatiotemporal pattern of endogenous GLAST expression, with minimal β-Gal activity in E9.5 telencephalon (g,h) but extensive staining by E10.5 (i,j); this pattern matches that previously reported for *Blbp*::Cre;R26R embryos [8]. Concordance between β Gal activity and GLAST^+ ^RG was consistently observed. At E12.5, GLAST is weak in septum (k'), and recombination in this region was similarly minimal (k). Conversely, GLAST and recombination are intense in the cortical hem (arrow in (l); higher magnification image of GLAST hem staining in (l')). Further, both Cre mRNA 1c) and recombination (m) in the spinal cord matched the dorsoventral gradient of endogenous GLAST in this region (Figure 1r). PH3 and GFP labeling in the E14.5 GE of Glast::Cre;Z/EG embryos showed that mitotic SVZ progenitors were recombined (n-q). (as observed in *Blbp*::Cre;R26R embryos [8], establishing that GE SVZ progenitors are RG-derive. Scale bars: 400 μm (a,c,g,i); 200 μm (k); 175 μm (b,d,j); 150 μm (h); 100 μm (m); 40 μm (f,n); 25 μm (l); 20 μm (e,o-q).

As either precocious or delayed Cre activity could obscure the true contribution of *Glast*^+ ^RG to neurogenesis, details on when and where Cre recombination first occurs embryonically is required. This information is also relevant for defining the timing of the neuroepithelial stem cell (NE) to RG developmental transition, marked by the presence in the latter of 'astroglial' characteristics. These have been defined as molecular features unique to astroglial cells in the postnatal brain and are proposed to include BLBP and GLAST [5,12,13]. We therefore stained embryonic sections to determine when these three 'astroglial' molecules first become detectable in the forebrain. At E9.5, neither GLAST nor BLBP could be detected in the forebrain whereas both were highly expressed in the hindbrain at this age (Figure 2a,c). By E10.5, both molecules were present in the forebrain and both exhibited similar higher-rostral, lower-caudal gradients of expression (Figure 2b,d). Furthermore, double labeling confirmed that both molecules are induced in the same cellular population (Figure 2e,f). These results demonstrate that cells with the molecular characteristics of RG are present in the forebrain by E10.5.

To determine when recombination occurs and how well correlated it is with endogenous GLAST, we crossed *Glast*::Cre mice to the Rosa26^LacZ^[14] and Z/EG [15] Cre reporter lines, which express β-galactosidase (R26R) or eGFP (Z/EG) after Cre-mediated recombination. Histochemical staining of *Glast*::Cre;R26R embryos demonstrated a tight concordance between the onset of recombination and onset of endogenous GLAST expression, with β-galactosidase activity and GLAST protein both becoming detectable in the forebrain at E10.5 (Figure 2a,b,g–j). Importantly, this spatiotemporal pattern of recombination also matches what was observed in *Blbp*::Cre;R26R embryos [8]. The fact that both endogenous expression and recombination driven by the promoters of two different RG genes are so similar strongly suggests that the NE→RG transition occurs between E9.5 and E10.5 in the forebrain. It should also be noted that the timing of recombination in *Glast*::Cre;R26R and *Blbp*::Cre;R26R embryos sharply contrasts with that driven by the neuroepithelial cell marker *Nestin*, as recombination in *Nestin*::Cre;R26R embryos is readily detectable throughout the CNS by E8.5 [16]. Thus, the data together provide both molecular and genetic evidence for the existence of a developmental transition that occurs by E10.5 in the forebrain, consisting of an earlier *Nestin*^+^*Blbp*^-^*Glast*^- ^NE stage and a later *Nestin*^+^*Blbp*^+^*Glast*^+ ^RG stage. Analysis of recombination in *Glast*::Cre;R26R embryos at later time points showed a similarly tight concordance between GLAST expression and recombination (Figure 2k–m), providing further support for the specificity of the *Glast*::Cre transgene and for the validity of the lineage tracing results.

### *Glast*^+ ^RG generate neurons throughout the brain

**Figure 3:**
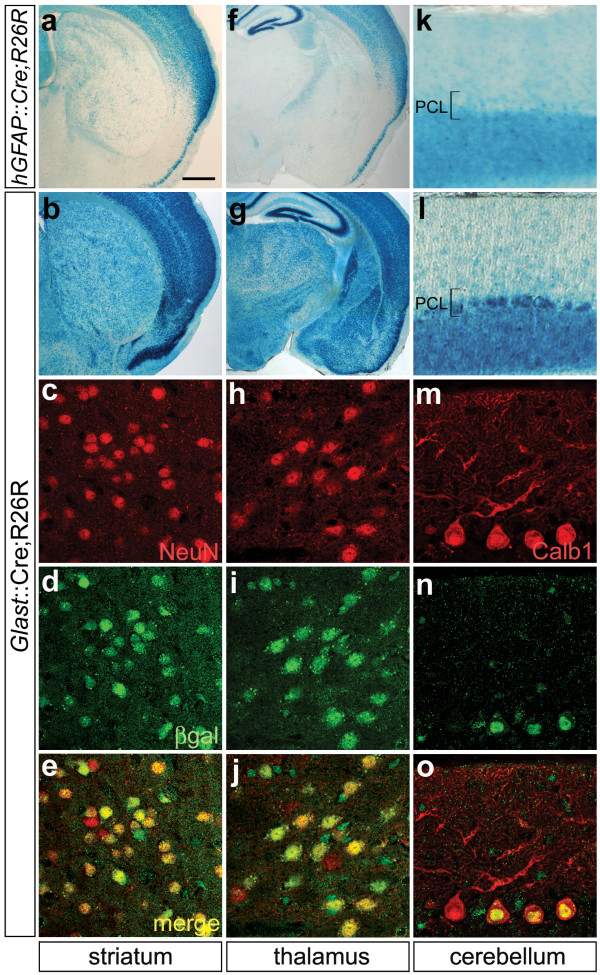
***Glast*^+ ^radial glia generate the majority of neurons in the brain.** (a-o) X-gal histochemical staining for β-gal activity (a,b,f,g,k,l) and immunofluorescent staining for NeuN (red) (c,e,h,j), β-galactosidase (green; d,e,i,j,n,o) and Calbindin (red) (m,o) in *hGFAP*::Cre;R26R (a,f,k) or *Glast*::Cre;R26R (b-e,g-j,l-o) adult mice. Regions immunofluorescently stained are striatum (c-e), thalamus (h-j) and cerebellum (m-o). In *hGFAP*::Cre;R26R mice, very few recombined neurons are present in the ventral telencephalon, diencephalon or midbrain, and cerebellar Purkinje neurons are unrecombined (a,f,k). PCL, Purkinje cell layer. In contrast, neuronal recombination was extensive throughout the brains of *Glast*::Cre;R26R mice (b,g,l) and double labeling confirmed that most neurons were recombined (c-e,h-j,m-o). The similarity of the *Glast*::Cre;R26R and *Blbp*::Cre;R26R lineage tracing results establishes that radial glia are the primary neuronal progenitors throughout the developing brain. Scale bars: 500 μm (a,b,f,g); 50 μm (k,l); 35 μm (m-o); 25 μm (c-e,h-j).

Adult *Glast*::Cre;R26R mice were analyzed to identify neurons derived from *Glast*^+ ^RG. For comparative purposes, we also examined recombination in *hGFAP*::Cre;R26R mice using the identical *hGFAP*::Cre transgenic line [9] as the Malatesta study [5]. Similar to previous reports [5,9], very few recombined neurons were observed in *hGFAP*::Cre;R26R mice in the ventral telencephalon, diencephalon or midbrain, and cerebellar Purkinje neurons were unrecombined (Figure 3a,f,k). In contrast, recombination in *Glast*::Cre;R26R mice was extensive in all of these brain areas (Figure 3b,g,l). Moreover, NeuN/β gal double labeling demonstrated that most neurons in these regions were recombined and, therefore, radial glial-derived (Figure 3c–d,h–j,m–o). As the *Glast*::Cre;R26R and *Blbp*::Cre;R26R tracing results [8] are essentially the same, these data establish that RG in all brain regions transit through a neurogenic stage of development and generate most neurons in the brain.

### *hGFAP *promoter activity is poorly correlated with RG differentiation in multiple brain regions

Why are the *hGFAP *lineage tracing results different from those using the *Blbp *and *Glast *promoters if all three promoters are cell-specifically expressed in RG? As neurogenesis precedes gliogenesis during development, one possibility is that the *hGFAP *promoter does not become active in some brain regions until after RG neurogenesis is complete. This idea is supported by two observations: eGFP expression in *hGFAP*::eGFP mice is undetectable in the GE until E14.5 [5]; whereas most *Blbp*^+ ^RG isolated from the E11.5 GE generated neurons in clonal cultures, most clones generated from cells isolated from E14.5 GE lacked neurons [8]. This latter result indicated that GE RG neurogenesis may be largely completed by E14.5.

**Figure 4:**
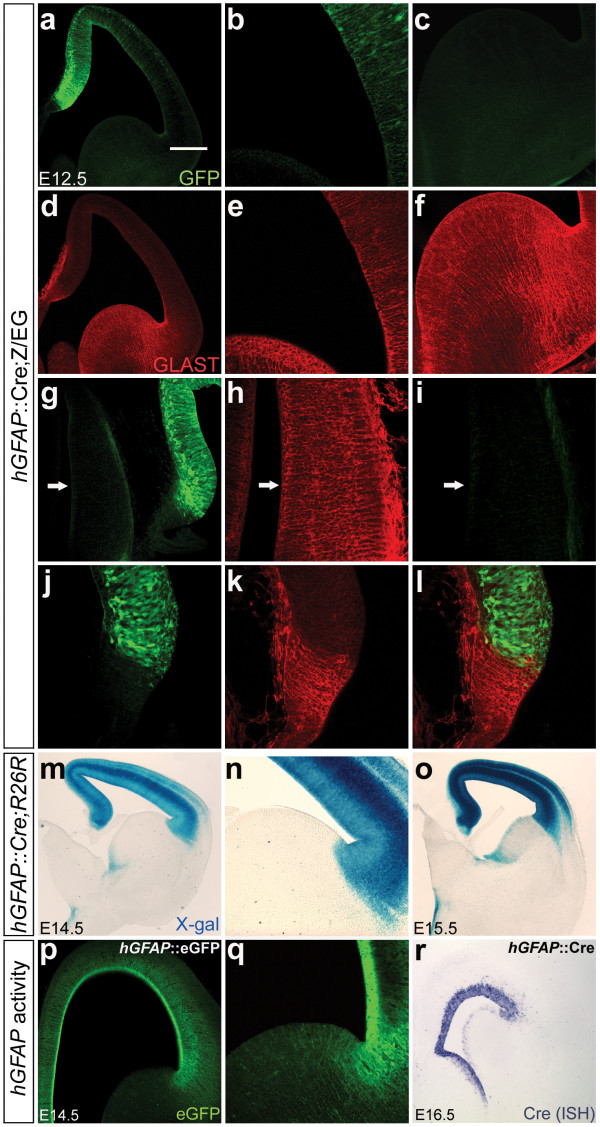
**The *hGFAP *promoter fails to drive recombination in fully morphologically differentiated radial glia (RG) of the ventral telencephalon and thalamus.** Immunostaining for eGFP (green) (a-c,g,i,j,l,p,q) and GLAST (red) (d-f,h,k,l), X-gal staining for β-gal (m-o) and Cre ISH (r) on hGFAP::Cre;Z/EG (a-l), hGFAP::Cre;R26R (m-o,r) or hGFAP::eGFP (p,q) embryos, at E12.5 (a-l), E14.5 (m,n,p,q), E15.5 (o), and E16.5 (r). Panels (b,c,e,f) show cortex and GE in (a,d) at higher magnification; panels (h,i) show thalamus in (g) at higher magnification, arrows in (g-i) mark identical spots in all three panels; panels (n,q) show higher magnification images of cortex and GE in (m,p). In contrast to the Blbp and Glast promoters, the hGFAP promoter is not active in forebrain prior to E12.5 (not shown). By E12.5, recombination is detected in cortical RG but absent from GE (a-c), thalamus (g,i) and cortical hem (j,l). Importantly, both BLBP (not shown) and GLAST (d-f,h,k,l) are highly expressed in these regions. Moreover, these unrecombined cells have molecular and morphological properties of fully differentiated RG cells (e.g. long radial processes coursing from VZ to pia). Even as late as E14.5, few GE RG are recombined (m,n), and significant recombination is not observed until E15.5 (o) when most RG in the region have completed neurogenesis. hGFAP promoter activity itself is not only delayed with respect to RG differentiation, but also weak ventrally compared to its dorsal activity (p,q,r). Scale bars: 300 μm (m,o,r); 200 μm (a,d,p); 100 μm (c,f,g,n,q); 60 μm (b,e,h-l).

To test the hypothesis that onset of *hGFAP *promoter activity is delayed with respect to the onset of RG differentiation, we determined when recombination first occurs in *hGFAP*::Cre;Z/EG and *hGFAP*::Cre;R26R embryos. Prior to E12.5, no recombination in the forebrain was observed in either reporter line (data not shown). By E12.5, extensive recombination had occurred in the cortex but was completely absent in the GE (Figure 4a–c). This result was striking as the GE VZ at this age is composed of fully morphologically differentiated BLBP^+^GLAST^+ ^RG (Figure 4a–f; note that all BLBP^+ ^and GLAST^+ ^cells in the E12 GE are RC2^+ ^[17], establishing that they are RG and not astrocytes). Similar results were observed in the thalamus (Figure 4h,i) and cortical hem (Figure 4j,l).

Analysis of the GE at E14.5 showed that some recombination had occurred medially, but the vast majority of VZ cells remained unrecombined (Figure 4m,n) and appreciable recombination could not be observed here until E15.5 (Figure 4o). Taken together, these results establish that *hGFAP *promoter activity is poorly correlated with RG differentiation in multiple brain regions, requiring several days after endogenous RG markers are first induced before becoming active. Direct examination of *hGFAP *promoter activity further illustrated this point: even though some expression can be detected in the E14.5 GE, significantly higher expression was observed in the cortex (Figure 4p,q). Moreover, weak ventral expression persisted as late as E16.5 (Figure 4r). This is the opposite of the endogenous RG markers BLBP and GLAST, which are more highly expressed ventrally at E14.5 and roughly equivalent at E16.5 [8] and further demonstrates that the *hGFAP *promoter does not accurately reflect normal characteristics of RG development in mice. Lastly, we note that previous work showed that all GE *hGFAP*^+ ^RG are also GLAST^+ ^[5] and that at E14.5 (when GE *hGFAP*-mediated recombination first occurs), all BLBP^+ ^RG are also GLAST^+ ^[17]. Thus, *hGFAP *does not define a unique BLBP^-^GLAST^- ^population of radial glia.

## Discussion

In this study we have clarified and defined two major aspects of RG development and function. First, we have shown that cells with the molecular features of RG are present in the forebrain from E10.5, establishing that the NE→RG transition commences in this region between E9.5 and E10.5. Second, we have solidified the concept that RG are the primary neuronal progenitors in the developing brain by showing that: most neurons in the brain are derived from *Glast*^+ ^RG; and in multiple brain regions, the heterologous *hGFAP *promoter is not active until several days after morphologically and molecularly defined RG first appear. Thus, the limited neuronal recombination driven by the *hGFAP *promoter is not indicative of regionally restricted potential as proposed [5,10,11] but rather a result of delayed *hGFAP *activity in certain RG populations.

As the recombination observed in Cre/*loxP *tracing reflects transcriptional activity of the promoter driving Cre, lineages can be traced from the time points when the molecular programs responsible for inducing promoter activity first become active. Results using endogenous mouse RG promoters such as *Blbp *and *Glast *therefore record lineages from the time when cells first become molecularly distinguishable as RG. The validity of this approach has been questioned by the suggestion that the presence of both molecular and morphological features are required in order to define cell types [11]. However, this idea does not take into account the fact that molecular changes necessarily precede and drive subsequent morphological changes. For example, the transcription factor *Hb9 *is required for normal specification of spinal cord motoneurons [18] and ectopic *Hb9 *expression is sufficient to induce motoneuron fate [19]. However, axon extension and innervation of skeletal muscle are not observed until at least one day after *Hb9 *transcription is induced [20]. Thus, although the presence of distinct morphology can allow cell types to be positively identified, their absence does not exclude a given identity during development since many morphological characteristics of differentiated cells appear only after the required molecular changes take place.

In addition to such reasoning, several lines of evidence support the idea that the NE→RG transition occurs in the forebrain by E10. First, expression of both *Blbp *and *Glast *becomes detectable in the forebrain by this time (Figure 2); thus, two different RG genes are induced at the same time. Second, Notch signaling promotes the RG phenotype [21,22] and *Blbp *is a direct Notch target gene [23]; therefore, induction of *Blbp *expression indicates that Notch-dependent RG differentiation has occurred. Finally, mice lacking *ErbB2*, a gene required for RG differentiation, begin showing impaired RC2 labeling in the telencephalon from as early as E9.5 [24] (some recombination restricted to the most rostral portion of the forebrain was detectable in E9.5 *Blbp*::Cre;R26R embryos [8]). Taken together, these data demonstrate that the molecular programs responsible for triggering RG differentiation are already active in some forebrain regions by E9.5 and induce broad expression of RG target genes by E10.5. They further suggest that an understanding of the mechanisms regulating the NE→RG transition will require analysis of molecular events occurring between these time points.

An important technical issue regarding Cre/*loxP *lineage tracing is that recombination efficiency is directly dependent upon cellular recombinase concentrations; this point must be considered when comparing results obtained using distinct transgenic or targeted lines. For instance, when four different promoters of varying strengths were used to drive Cre and compared in embryonic stem cells, recombination efficiency was directly correlated with promoter activity: the strongest promoter drove the highest levels of recombination [25]. Furthermore, even when the identical promoter is used, recombination efficiency can vary dramatically according to copy number. For example, merely doubling the copy number of a *β-actin*::Cre transgene (from one to two copies) resulted in a 10-fold increase in the number of recombined cells in the brain [26]. For these reasons, caution must be used when interpreting negative results in lineage tracing experiments as a lack of recombination could reflect weak promoter activity rather than restricted progenitor potential. Indeed, differences in copy number likely explain why subsequently generated *hGFAP*::Cre transgenic lines yielded higher levels of neuronal recombination [27] than observed using the original line [5,9]. It will be important to keep these issues in mind when comparing lineage tracing results in which the same genomic locus was either targeted (that is, knockin) or used as a transgene. Whereas knockins can, at most, provide two targeted alleles, tens to hundreds of copies are typical for transgenics [28]. Therefore, in many cases, transgenics may be more likely than knockins to drive recombination at the time when transcription commences and consequently should give a more accurate picture of the progeny derived from a given molecularly defined progenitor population.

Although the onset of *Blbp *and *Glast *transcription distinguishes RG from *nestin*^+^*Blbp*^-^*Glast*^- ^NE, the available evidence indicates that, as a population, *Blbp*^+^*Glast*^+ ^RG themselves transit through multiple distinct developmental stages. For example, the *hGFAP *promoter does not drive any recombination in cortex prior to E12.5 (data not shown). As rodent *Gfap *is not normally expressed so early in RG neurogenesis, it is possible that the timing of *hGFAP *activity in mice merely reflects the peculiarities of a heterologous promoter. However, it is alternatively possible that a fundamental transition occurring at E12.5 upregulates *hGFAP *activity; if so, the latter stage might represent the terminal phase of RG generation of layer VIb neurons as only about 30% of these neurons were recombined in *hGFAP*::Cre;R26R mice [5]. It is also well known that RG ultimately cease generating neurons and switch to production of glia; although several pathways have been implicated in promoting gliogenesis (for example, Jak-STAT signaling [29] and NFI genes [30]), a comprehensive description of the mechanisms involved is lacking and requires transcriptional profiling and comparisons between neurogenic and gliogenic RG. Steps in this direction have been taken by transcriptome analyses of FACS purified RG whose neurogenic potential was determined by *in vitro *clonal analyses [31]. However, given that progenitor neurogenic potential *in vivo *can differ from that observed *in vitro *[32], subsequent studies might yield additional insights by defining potential on the basis of either *in vivo *transplantation or genetic lineage tracing. Accordingly, our lineage tracing data provide *in vivo *evidence that *hGFAP *promoter activity commences in ventral telencephalic RG only after neurogenesis is largely completed and thus distinguishes a later *Blbp*^+^*Glast*^+^*hGFAP*^+ ^gliogenic stage from an earlier *Blbp*^+^*Glast*^+^*hGFAP*^-^neurogenic stage in this region. This is significant as it enables direct *in vivo *comparisons of neurogenic and gliogenic ventral telencephalic RG via FACS purification and microarray analyses using *hGFAP*::eGFP and *Blbp*::eGFP or *Glast*::eGFP transgenic mice. Such studies should facilitate efforts to more thoroughly determine the molecular mechanisms that control progenitor potential and fate specification.

## Conclusion

These data establish the major neurogenic role of RG in the developing CNS and genetically distinguish an early neurogenic *Blbp*^+^*Glast*^+^*hGFAP*^- ^stage from a later gliogenic *Blbp*^+^*Glast*^+^*hGFAP*^+ ^stage in the ventral telencephalon.

## Materials and methods

### Mice and BAC transgenesis

All protocols were approved by the Rockefeller University IACUC. *Glast*::*Cre *BAC transgenic mice were generated using homologous recombination to insert a Cre-SV40polyA cassette at the site of the endogenous ATG (exon II, BAC clone RP23-63O21). Transgenic FVB/N mice were generated using standard procedures [28]. R26R^lacZ^, Z/EG, *hGFAP*::Cre, and *hGFAP*::eGFP mice were obtained from JAX (Bar Harbor, Maine, USA). *Glast*::eGFP mice were obtained from GENSAT (New York, NY, USA). The BAC clone used to generate *Glast*::eGFP mice is the same as that used for the *Glast*::Cre mice and the eGFP-polyA cassette was targeted to the identical locus in the BAC as the Cre-polyA cassette. Analysis of endogenous gene expression was done using C57BL/6J (JAX).

### Tissue processing and analysis

Tissues were either immersion fixed (embryos) or perfused (postnatal/adult) with 4% paraformaldehyde, cryoprotected in 15% sucrose in phosphate-buffered saline (PBS), and cut on a cryostat (15 μm) or vibratome (75 μm). Cryosections were dried at room temperature for 2 hours, a step that improved signal (insufficient drying was responsible for the previous inability to detect BLBP protein in the E10.5 forebrain [8]). Primary antibodies used were: rabbit α-BLBP (1: 1500); rabbit α-β gal (1:500; ICN Santa Ana, CA, USA)); mouse α-Calbindin (1:500; Swant, Bellinzona (Switzerland)); rabbit α-Cre (1:5000 and TSA amplified; Novagen, San Diego, CA, USA); goat α-GFP (1:500; US Biological, Swampscott, MA, USA); guinea pig α-GLAST (1:5000; Millipore, Billerica, MA, USA); mouse α-NeuN (1:200; Millipore, Billerica, MA, USA); rabbit α-phosphorylated histone H3 (PH3, 1:250; Millipore, Billerica, MA, USA). Alexa 488-coupled secondary antibodies were obtained from Molecular Probes, and Cy3-coupled secondaries were from Jackson Immunoresearch (West Grove, PA, USA); both were used at 1:750. Sections were preblocked in 5% donkey serum, 0.1% Triton X-100 in PBS and incubated with primary antibodies overnight at 4°C. After washing, sections were incubated with secondary antibodies (generated in donkey) for 2 hours in the same solution at room temperature. Confocal imaging was done on an LSM 510 Axioplan (Zeiss). For X-gal histochemical staining, tissues were incubated at 37°C in X-Gal solution: 1 mg/ml X-gal (Sigma, St. Louis, MO, USA)), 20 mM K_3_Fe(CN)_6_, 20 mM K_4_Fe(CN)_6_, 2 mM MgCl_2 _and 0.02% NP-40 in PBS.

### Non-radioactive ISH

Riboprobes to detect Cre mRNA were generated using T7 polymerase off PCR amplified template DNA generated with the following primers: forward, CAAGCTCGAAATTAACCCTCACTAAAGGGgtccaatttactgaccgtacacc; reverse, AGAGAGCGGTAATACGACTCACTATAGGGCctaatcgccatcttccagcag. Probe hybridization and development was done using standard protocols.

## Abbreviations

BAC: bacterial artificial chromosome; BLBP: brain lipid binding protein; CNS: central nervous system; E: embryonic day; eGFP: enhanced green fluorescent protein; GLAST: glial high affinity glutamate transporter; hGFAP: human glial fibrillary acidic protein; ISH: *in situ *hybridization; NE: neuroepithelial stem cell; PBS: phosphate-buffered saline; PH3: phosphorylated histone H3; RG: radial glia; VZ: ventricular zone.

## Competing interests

The authors declare that they have no competing interests.

## Authors' contributions

TEA performed all experiments and wrote the manuscript with input from NH.
